# Etiology of Shoe Boil1Read before the Twelfth Annual Meeting of the New York State Veterinary Medical Association, at Brooklyn, September 9 and 10, 1902.

**Published:** 1902-11

**Authors:** G. J. Goubeaud

**Affiliations:** Brooklyn, N. Y.


### ETIOLOGY OF SHOE BOIL.[1]

By G. J. Goubeaud, D.V.S.
BROOKLYN, N. Y.

About two years ago I read an original paper before the New York County Veterinary Medical Association, treating upon etiology of shoe boils, or, as more properly termed, ulnar fibroma. It was prepared after several years of study, thought, investigation, and experiment. Not being easily discouraged by adverse criticism, I have continued investigating up to the present time.

It is with the earnest desire of, as it were, forcing the issue, that I lay this, to me, interesting subject before this association, a society composed of the foremost thinkers and advanced veterinarians of the country. The evening I read the paper before the New York County Veterinary Medical Association I did not possess as much as one friend or one who believed in my assertions.

In the discussion that followed I was plied with more questions than ten quick thinkers and rapid talkers could answer. Every

[1] Read before the Twelfth Annual Meeting of the New York State Veterinary Medical Association, at Brooklyn, September 9 and 10, 1902.

one opposed me, and I left that meeting more determined than ever that I was right, because no one advanced a common-sense argument, made a reasonable assertion, or presented a rational view to upset my opinion. But since then I have received expressions of opinion which are in accordance with those which I hold.

I lay this subject before the members of this Association to get an expression of opinion.

First, I wish to know if I am right. And if so, then I have added my mite in advancing the interests of veterinary science; and if wrong, then I have simply been mistaken. I know that it is with no little difficulty some of us cast aside fixed ideas, cherished traditions, the teachings which we received, ideas formed, and the impressions conveyed to us in our college days. It is with a certain amount of timidity that I present this paper, and I hope to have your indulgence. It is not the intention of the essayist to enter minutely into the pathology of this affection, or describe the various lesions which the injury can produce (for such it primarily is), or the complications which may arise; nor its therapy. Aside from prevention the etiology alone will be considered.

The text-books, veterinary authorities, professional and lay writers, and those with whom I have conversed, claim that the shoe or the hoof is the offending agent. The larger number claim that the shoe causes this affection.

We will first take up the shoe as the first cause. The shoe causes shoe boil, or it does not. The animal is either improperly shod, the heels of the shoe are too long, or they are purposely made long in order to correct some abnormal condition of the foot or irregularity of the animal's gait.

The animal while in the recumbent position rests the elbow upon the heels of the shoe, thus injuring the skin and structure beneath, resulting in the characteristic tumor, or it drops heavily upon the ground while assuming the recumbent position. The heels of the shoe come in violent contact with the elbow, and in this manner the tumor is developed.

Now as a rational conclusion, a result can exist not produced by a cause. How can a result take place which is produced by a cause that does not exist, and has no existence, or, in other words, how can a shoe cause shoe boil when the horse did not wear shoes at the time of the development of the tumor? I have seen colts that never had a shoe upon their feet have these tumors, and I have seen horses without shoes turned loose in a box-stall, and horses turned loose without shoes in a pasture lot, develop ulnar tumors. Others have observed the same occurrences, and still it seems to make no difference, shoe or no shoe, these animals develop the affection.

I have seen horses wearing rubber pads have a swollen condition of the elbow present itself over night. In two instances the rubber pad was employed as a preventive measure, and still there were recurrences in both cases. I have seen horses wearing the half shoe develop tumor of the elbow. Now, how is it possible for the horse to develop these tumors caused by the shoe when the horse does not and did not wear shoes at the time of the occurrence of this affection?

Some claim that the foot causes this abnormality, and I will attempt to disprove the correctness of this assertion, although it will be a much more difficult task.

We will suppose that an animal does rest the elbow upon the foot, which part of the hoof I do not think makes any difference. We will suppose that a weight, say fifty pounds, rests upon the foot, and the pressure of the same amount is placed upon the region of the elbow. Does it seem reasonable to suppose that an animal with a healthy brain will lie in that position? Does it seem reasonable to think that an animal will lie half an hour, or an hour, or perhaps longer, without causing extreme pain and discomfort? Does it seem reasonable to think that an animal will so lie upon the skin of the elbow and the structures beneath, which are very sensitive, and that it will injure them so severely without changing its position, and that the necessarily painful and uncomfortable position will not be changed before any damage has been done? As to the foot, will an animal stand that amount of pressure upon the hoof and its sensitive structure? I say, no.

Compress a horse's foot to about a twenty-five pound pressure, and I can assure you that he will rebel against any such treatment the moment the test is applied, and it will not be necessary to wait one-half hour for the result; and again, the usual result of pressure applied to sensitive parts is lowering of the vitality, destruction of the parts, pus formation, and sloughs. I ask how often does this happen? I can answer, very seldom.

For the purpose of a better understanding of the subject under consideration it will be necessary for me to divide ulnar hygromas into two classes—the acute and chronic. This will enable me to better explain the reason for their repeated occurrence and their non-occurrence.

By the acute hygroma I mean that condition of the elbow when it is first seen. Here we find an animal with a tumor of the elbow; it is hot, painful upon pressure, more or less œdema of the skin, the animal has more or less lameness. His condition receives the proper treatment, and in due time it disappears. There are no recurrences of the tumor, the severity of symptoms and size of the tumor depending upon the intensity of the cause producing it.

By the chronic form I mean when we have recurrences, or the cause producing this condition is not removed, and the result is an organized tumor. It is removed by surgical procedure, and still there are recurrences. I have seen cases that, in spite of treatment, developed into tumors, even after so-called preventive measures were applied; and in order to describe why they developed it will be necessary for me to enter into a discussion upon pathology, which I have no intention of doing.

For the time being we will turn our attention to the cow. Cows have these tumors, but they are rare, which fact can be explained by the difference in the anatomical structure of this region. I have seen but one case in a cow, and this individual animal had a well-developed hygroma of two-years' standing. A common condition of the elbow, which can be seen in cattle as well as in horses, is a thickened appearance of the skin upon the outside of the ulnar region, sometimes of a scabby appearance, or a large ulcerated wound will be found with the edges much thickened. This condition in cattle is found in milch cows confined in narrow stalls. The animal while lying in the recumbent position extends the foreleg on the side upon which it lies. It inclines the body to one side, propped up by the side of the stall or partition. The animal in extending the foot will strike the floor of the stall sufficiently hard to injure the skin, but no further, and in time we have the appearance of this condition presenting itself which I have described above.

The reason for the rarity of a well-defined tumor being found upon the elbows of cattle is that the sternum comes in contact with the floor, and the elbows cannot come in touch with the ground. The only manner in which the affected part can become injured is while reclining sideways with the side braced up by the partition. And again, horses and cattle do not assume the standing position alike. I say that cows do not wear shoes, they cannot lie upon their feet, and still, what causes this condition. Take the dog—especially those of the large variety. This is a common ailment, yet they wear no shoes. They cannot lie upon their feet; still, what causes these tumors and their recurrence, even after surgical removal?

Take the camel. Its position while recumbent is unlike that of the cow and the horse, but while attempting to rise is entirely different from that of the horse and somewhat similar to that of the cow. Elbows, knees, patellar region, os calcis and foot rest upon the ground, and in these parts there is a well-defined thickened condition of the skin, almost bordering upon the appearance of a tumor. I never saw a tumor in these animals, yet they wear no shoes; they cannot lie upon their feet. Now, what causes this thickened condition of the skin and the absence of a well-defined tumor?

The same can be said of the giraffe. The buffalo is similar to the cow; elk the same. The bear, fox, tiger, and lion are like that of the dog. I saw a well-defined ulnar tumor in a lion the property of a circus. These animals cannot lie upon their feet; they wear no shoes, and still what causes these growths?

Even man is not exempt from this affection. In this individual case, which I saw in a hospital, the tumor presented all the typical appearances of an ulnar fibroma caused in falling, by striking the elbow upon the ground. This man was an epileptic.

I have read reports in the different medical journals in reference to the tumors. The authorities agree that they are the result of an accident, such as falling, striking the elbow upon some solid body, etc.

And again, take the horse with the chronic shoe boil, and admitting that the elbow and foot can come together, the foot can be dislodged most readily with one finger, which I have done numbers of times and in the presence of witnesses. Now, if there was any pressure it would require more than the strength of one finger to dislodge the foot. Again, some claim that the motion of the animal's body causes friction, thus producing this affection.

When an animal assumes the recumbent position, it is with the object of resting its body, not to keep in motion; and secondly, friction produces burning, chafing, scalding, and removal of the hair and skin. Not one of these conditions can be found to exist.

If an animal lies upon the right side he appears to place the weight upon the elbow and foot. Now, examine the right side, and you will find the side upon which most of the weight rests, and you will notice that the foot is either to the right or left of the elbow, and not resting one upon another, however, much they may appear to be. And again, take the vast majority of horses that do not possess this abnormality. They assume the recumbent position with the knees and hock flexed, the body inclined to one side. It is the normal position and a natural condition not usually conducive to produce this affection. Take for example, an animal resting in the recumbent position upon the right side. Note its position. The left knee is flexed, the foot and elbow are in almost direct contact, the elbow apparently resting upon the heels of the foot. Now grasp the whole foot with the hand, and you will find that it is most readily and easily dislodged, showing that there is little or no weight placed one upon the other, and because this is the position which is the normal one for an animal to assume we immediately accuse the shoe or the hoof as the offending agent, simply because we see the elbow and the foot in juxtaposition. We come to the conclusion that the hoof, or shoe, or both, cause this abnormality.

It is true that we sometimes find the hoof either to the right or to the left of the elbow, but this is not common in horses. We will consider the side on which most of the weight rests, as, for example, that of the right side, where we find the hoof in this case; the left can be readily seen, but the right is hidden from view by the animal's sternum and pectoral region, and also the bedding. Do we find the foot resting upon the elbow? I say, no. Here we find the foot in the axillary space, toe turned inward with the elbow resting slightly upon the transverse surface of the fleshy portions of the heels. The weight upon the parts is distributed in this manner: first to come in contact with the floor is sternum, anterior face of knee, anterior right lateral face of the shin-bone, phalanges, anterior left lateral face of the hoof. To dislodge the hoof upon this side and bring it forward or outward is a difficult task to do. It requires some strength. The cause of this is that the weight is not upon the hoof but upon the structures which I have just described.

A raw surface is very rarely seen upon the internal face of a shoe boil. It is only seen upon the centre in the upper portions or upon the outside of the tumor.

The position which I have found animals assume, and which I firmly believe to be the cause of this affection, is that an animal will, while attempting to assume a standing position, and which he will necessarily have to do, is to extend the foot and flex the knee, thus forming an arch with the foot and elbow resting upon the ground. While attempting to assume the standing position he strikes his elbow forcibly upon the floor or ground, thus producing various lesions. In the course of my investigations I have seen this occur twice, the animals evincing symptoms of acute pain and lameness the following morning.

I also noticed that those which were predisposed to this affection were animals that assumed this position when recumbent, and they slept with the head between the knees and the nose resting upon the floor. They usually rested with one side of the body close to the side of the stall. It will often occur when the animal becomes frightened and arises suddenly. It will then strike its elbow upon the floor in a forcible manner, thus injuring it. I know of one instance where this occurred. I removed a splinter of wood, about the size of a match, from the skin of the elbow region. The animal was very lame for a week, after which the lameness gradually passed away, and he made a good recovery with no recurrence.

I have attempted in this short article to express my views as clearly as possible, and I hope in the discussion which follows that I will be able to defend the ideas which I now hold.

